# Monitoring the referral system through benchmarking in rural Niger: an evaluation of the functional relation between health centres and the district hospital

**DOI:** 10.1186/1472-6963-6-51

**Published:** 2006-04-12

**Authors:** Paul Bossyns, Ranaou Abache, Mahaman S Abdoulaye, Hamidou Miyé, Anne-Marie Depoorter, Wim Van Lerberghe

**Affiliations:** 1 Belgian Technical Cooperation (BTC), Brussels, Belgium; 2 Ministry of Health, Niamey, Niger; 3 Ministry of Health, Ouallam District, Niger; 4 Ministry of Health, Niamey, Niger; 5 Free University Brussels, Brussels, Belgium; 6 WHO, Geneva, Switzerland

## Abstract

**Background:**

The main objective of this study is to establish a benchmark for referral rates in rural Niger so as to allow interpretation of routine referral data to assess the performance of the referral system in Niger.

**Methods:**

Strict and controlled application of existing clinical decision trees in a sample of rural health centres allowed the estimation of the corresponding need for and characteristics of curative referrals in rural Niger. Compliance of referral was monitored as well. Need was matched against actual referral in 11 rural districts. The referral patterns were registered so as to get an idea on the types of pathology referred.

**Results:**

The referral rate benchmark was set at 2.5 % of patients consulting at the health centre for curative reasons. Niger's rural districts have a referral rate of less than half this benchmark. Acceptability of referrals is low for the population and is adding to the deficient referral system in Niger. Mortality because of under-referral is highest among young children.

**Conclusion:**

Referral patterns show that the present programme approach to deliver health care leaves a large amount of unmet need for which only comprehensive first and second line health services can provide a proper answer. On the other hand, the benchmark suggests that well functioning health centres can take care of the vast majority of problems patients present with.

## Background

Referral is the weak link in the organisation of many district health systems in rural Africa. In theory health centres and district hospitals should complement each other: whoever can be treated adequately at health centre level will be treated there, and the referral system will ensure that all others are referred to the district hospital in a timely fashion [1-6]. The criteria for referral are supposed to be purely medical, and objective, in the interest of the patient. This appears straightforward, but everything indicates that referral systems are usually dysfunctional [7-9]. Studies on maternal health in particular readily blame inadequate referral systems [10-19].

In urban areas often many false positive referrals are observed: pressured by patients, nurses at the first contact level readily refer a majority of patients to overcrowded hospitals – the latter having to deal with typical first line health problems as well as sophisticated surgical problems [20,21]. The health centre does not act as a gatekeeper.

The opposite happens in rural areas: referrals are too few and come too late. Patients, even those who attend a health centre fail to reach largely underused hospitals. This represents a nagging problem for managers: issues as diverse as provider behaviour, cultural barriers, financial constraints and criteria for referral need to be addressed [22-32]. Distance and transport problems are often critical [33-36].

In an ideal world there would be no false negatives and no false positives among those referred. False negative referrals are not registered and may die in the village without anybody noticing the fact that maybe the patient did consult a health centre previously. False positives on the other hand are difficult to define: what is a legitimate reason for referral for the patient or for the nurse does not necessarily correspond with the doctor's opinion. Hence both false positive and false negative referral cases are difficult to grasp through routine data or rational guidelines.

Ultimately, one would like to know whether one is referring too many or too few people. Monitoring referral rates – the proportion of patients at the first line referred to hospital, or, more appropriate, a population-based referral rate – is complicated by the fact that there is no such thing as a 'good' and universally valid referral rate. One should expect a wide variation between and within countries as the frequency of referral depends on many factors such as poverty, population densities, the density of the network of health centres, the availability of transport and organization of emergency transport, as well as on the performance and facilities of the health system. Even in rich countries, referral rates vary widely [37-40]. They are often determined in an urban environment. There are but few studies in less-developed countries. WHO's Integrated Management of Childhood Illness' (IMCI) programme mentions referral rates for children that range from 3 to 27% [41,42]. In an African context optimal referral rates ranging between 5 and 8% have been recommended [20]. These figures are merely based on field observations most often in urban areas, not on experimental benchmarking.

Niger's very low hospitalisation rates suggest that referral is a real problem in this poverty stricken country. A number of studies confirm this, particularly in the case of emergencies [43-45]. Niger monitors its referral rates, but no matter how sophisticated the counting of referrals may be, the numbers need interpretation and contextualization. This study sets a benchmark for referral frequency in order to allow interpretation of referral rates in rural Niger, thus rendering them intelligible for diagnostic and planning purposes.

## Methods

The estimation of the benchmark for referral rates for curative services at rural health centres was done by documenting the frequency and characteristics of referrals made under conditions of strict application of clinical guidelines. This exercise took place in Ouallam district, Niger. It is a typical rural Sahel district with 270,000 inhabitants dispersed in a vast territory of 22,000 km^2^. As for the rest of rural Niger, people have difficult access to first line health care. Only 26 % of its population lives in an area of 5 km from a health centre. Geographical inaccessibility combined with poverty explains the low curative first line care utilisation rates: an average of 0.21 contacts per person per year over the past 5 years for the whole population and of 0.81 if only the population within an area of 5 km from a health centre is considered. This utilisation rates fall within the range recorded from 11 out of the 39 rural districts of Niger: between 0.14 and 0.46 for the total population and between 0.38 and 0.89 for the population within 5 km from a health centre.

Clinical guidelines under the format of decision trees are a widespread tool to organise first line curative services in Africa. In Niger, nurses at health centre level have been asked to work with clinical decision trees since 1984. They were an adapted version of those already tested and in use for several years in Cameroon. In 1999, these guidelines were revised by an expert committee of specialist medical practitioners of the national hospital, national and international public health specialists familiar with curative care in rural districts and district medical doctors. Some missing guidelines, for example for burns, were introduced and more attention was paid to psychiatric and psycho-somatic symptoms. IMCI guidelines, slightly adapted for Niger to be more sensitive in identifying and referring malnourished children were integrated in the guidelines. A field test of the clinical guidelines led to a few minor changes.

Routine data on referral rates can be compared to the benchmark resulting from this controlled application. Assuming that the guidelines, correctly applied, minimize false positives and false negatives, this comparison gives an indication of the extent of under- or over-referral under routine circumstances.

Three final year medical students were selected as field researchers. They had better clinical expertise for a correct and critical interpretation of the decision trees than the nurses who normally run health centres in Niger. Researcher A applied the guidelines in three health centres during one month each, researcher B applied them in a fourth health centre during 2 months and researcher C during 3 months in a fifth health centre (Table 1). They had received extensive training on the use of the clinical decision trees in the urban health centre of the district. The research periods covered the dry and wet season to avoid a possible seasonal influence on the type of demand.

In their assignment to the rural health centres, the researchers saw all curative patients during clinic hours, as well as all emergency cases outside normal working hours. The total number of these patients was 3,905. Any deviation from the guidelines was documented and was restricted to cases where the researcher felt that application of the instructions might endanger the patient's life. Reasons for referral were recorded. A priori healthy clients presenting in preventive clinics were not considered: referrals based on risk factors only as is classically the case in antenatal care (ex. multiple pregnancy or previous caesarean section) were not considered as curative referrals for this study in concordance with the definition of the national health information system concerning curative referrals. But if frank pathology needing referral was observed in pregnant women that needed referral (ex. bleeding placenta previa), such patients were considered curative cases and were included in the referral statistics.

The referrals resulting from this strict application of the decision trees were considered as a golden standard. Clinically skilled and specifically trained to apply the standardized instructions, the medical students would avoid false negative or false positive referrals.

Referral rates were calculated as number of referrals per 100 new patients, distinguishing between emergency and cold referrals. Repeat visits for the same illness, were not counted in the denominator as the patient was already in for the same condition. Emergency referrals were defined as those where the field researcher called for an ambulance (care having been taken to make this a realistic option); the others were classified as cold referrals. A referral was categorised as "accepted" if the patient expressed agreement and intention to go to the district hospital; a referral was classified as "complied" if the patient effectively presented to the hospital. These two categories were used to calculate an "acceptance" and a "compliance" rate.

The benchmark was then used to interpret referral rates in the rural and urban health centres of 2 regions of the country, totalling 11 districts out of the country's 42. For the period 1995 to 2002, yearly referral rates for districts were extracted from the routine health information system of the districts. These routine data were not always reliable and complete. In total 11 of these yearly referral rates were either not available or so obviously unreliable that they were excluded from further analysis.

## Results

### Benchmark referral rate

One-third (36%) of the 3,905 patients were younger than 5 years of age; half (49.6%) were female. Strict application of the clinical guidelines resulted in 47 emergency evacuations and 49 cold referrals: 2.5% (C.I. ± 0.5 %) of the 3,905 patients seen (Table 1).

Although 85% of the 47 patients for whom emergency referral was proposed expressed acceptance of the decision, and although emergency transport was made available, only 26 (55%) actually complied and were evacuated to the district hospital (Table 2). Nearly all the instances where emergency evacuation eventually did not take place were children under 5. At least 8 died in the hours or days after referral was proposed. Emergency transport had been made available, but lack of money to pay the ambulance fees was a major obstacle.

#### Acceptance and compliance rates of referral proposals

Non-emergency referrals were easily agreed to by 90% of the patients, but this did not mean that the referral advice was effectively complied with. Compliance was difficult to monitor in this group since patients had different options: the district hospital or the hospital in the capital of the country. Those who refused to be referred included patients with obvious and severe pathologies: for example, 3 had progressive paralysis of the legs with a painful deformation of the vertebral spine (probably cases of Pott's disease), and one had a severe bilateral eye infection with blindness. Compliance with emergency referral proposals was particularly low for children: 62% of emergency referral proposals for children below 5 years were not complied with.

#### Reasons for referral

Reasons for referral were quite diverse (Table 3). Twenty percent of the referrals were children under 5, though they represented 36% of the patient population, which gave a child-specific referral rate of 1.4%. There were only two cases of severe malnutrition and nine of severe respiratory infection.

Gynaeco-obstetric cases represented 18% of the referrals: acute obstetric emergencies such as foeto-pelvic disproportion (2), placenta preview (3) or shoulder presentation (1), but also four cases of third degree prolapsed uterus, one vesico-vaginal fistula and four women with problems of sterility.

The 19 surgical cases included six men with urinary retention and nine cases of serious trauma. Among the 41 general medicine patients were 6 cases with tuberculosis of which 4 Pott's disease and one frank haemoptysis.

### Routine referral rates in Niger's districts

**Figure 1:**
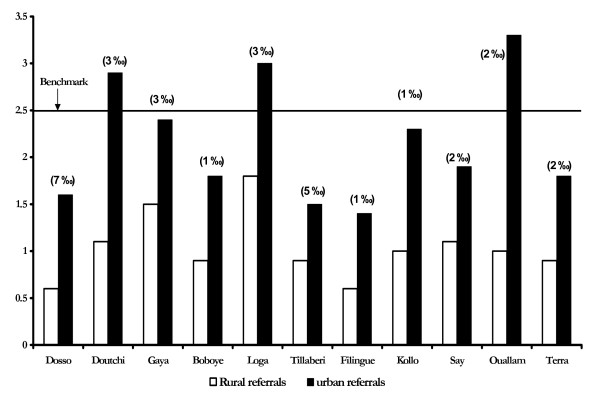
Observed rural and urban referral rates compared to the ”strict guidelines application„ benchmark. Figures in brackets represent population-based yearly hospitalisation rates in the district.

In 11 health districts, referral rates were extracted from the annual reports compiled routinely in each district. Rural referral rates were systematically lower than those in urban areas (Figure 1) and far below the 2.5% benchmark. In some districts, referral rates in urban areas were higher than the benchmark, but even in these settings, where distance and transport costs are no major barriers, referral rates were often well below 2.5%.

With such low referral rates and the major accessibility problems in Niger, it is not surprising that hospitals are largely under-utilized. Only in one of the 11 district hospitals there were more than five hospitalisations per 1000 inhabitants per year (Dosso, 7‰, Figure 1). In six districts there were two or even less.

Dosso has a regional hospital with specialist services, with good direct access for its own local urban population and even for the neighbouring districts along the national road. Given Dosso's low referral rates, it is clear that hospitalised patients had largely bypassed the health centres.

Only 5 of the 11 hospitals under study offered a full range of basic hospital care including surgery and blood transfusion. Among these five, only Dosso had 24 hour coverage for surgical care. With only one doctor with surgical skills, the other four could not offer a continuous service.

## Discussion

With the present case mix of patients presenting at the rural health centres in Niger, implementation of the existing clinical guidelines leads to a referral rate of around 2.5% of all new patients. Just under half of those would be emergency referrals. The three field researchers obtained similar results, which indicates that the referral rate benchmark was rather stable. There are no indications that the case mix was significantly different in other rural settings in Niger. Therefore the benchmark seems relevant for the rural areas in the country. Simple extrapolation to other countries is less obvious. The benchmark is sensitive to patient mix and utilisation rates which differs much between countries. However, the whole exercise cost less than 1000 $ US and can easily be reproduced in other settings.

Referral rates and compliance were lower for young children and this was associated with excess mortality. The child-specific referral benchmark was a mere 1.4%, well below the 27% IMCI proposes for Niger [42]. There is clearly a case for revisiting IMCI benchmarks, also in order not to damage the credibility of the entire programme with referral requirements that would be perceived as unrealistic and unacceptable by both the health centre staff and the population [46]. Low referral compliance resulted in a hidden mortality in the district that could have been at least partially avoided: patients actually did attend the health service, but the system failed to respond adequately. Even for emergency cases (55%), compliance was low, resulting in excess mortality. High acceptance, but low compliance rates indicate there is an unmet, though felt, need.

The benchmark is more likely to be an under- rather than an over-estimation of need for referral. The referrals that were made cannot be dismissed as false positives – there is little scope for frivolous over-consumption in a context where the cost -financial and cultural- of referral is so high. This implies that the deficits compared to the benchmark observed in the districts are likely to be underestimated as well.

The referral needs indicated by the benchmark were surprisingly low compared with what is indicated by the literature. Certain frequent pathologies such as severe malnutrition were hardly ever referred (2 cases over an 8-months period of observation, out of more than 3000 patients) maybe because guidelines were not entirely correctly applied at all times, but also because malnutrition without associated frank pathology is not a reason for consulting in rural Niger. Under-fives' preventive clinics in Niger are often of such quality that even at that level, malnutrition is seldom diagnosed and even less referred. However, apart from the under-representation of severe malnutrition among referrals, there were no indications that the benchmark was the result of excessively specific guidelines: if that had been the case the field researchers would have communicated this and would have proposed referrals disregarding the clinical instructions.

The low referral rates are a reason to invest further in comprehensive health centre care close to the population [47]: health centres in Niger have the potential to take care of 97 % of those who consult them.

The large variety of referral reasons shows how crucial comprehensive services at the district hospital, with a large spectrum of interventions, are to respond to people's health needs. For example, there were as many gynaecological reasons for referral as obstetric ones. Four women with third degree prolapsed uterus and 4 with long-term sterility complied with the referral. This is exemplary of pathologies that have a great social significance (in Niger both conditions lead to divorce or the acceptance of a second wife), but are often neglected by the classical programmes that give support for a limited number of -supposedly crucial- pathologies and ignore the broad scope of (perceived) important problems in the population.

Analysis of the reasons for referral shows important gaps in the health care provision, either at health centre or at district hospital level. There are problems for which no programmes are defined and for which the present health care network does not provide answers either. For example, among this limited sample of patients there were four cases of relatively advanced Pott's disease, whereas there is only one (expatriate) orthopaedic surgeon in Niger who can perform corrective surgery for a collapsed vertebra. One other patient complied with a referral over 300 km to the national hospital for toothache – a situation that district hospitals are not equipped to deal with. Six men were referred for urinary retention, but none was offered any other care than a permanent catheter. The fact that referral for severe malnutrition is hardly ever done or complied with, may well be because people know that the district hospital has no means to deal with the problem rather than for reasons of acceptability or cost.

Compliance rates in this study may have been higher than under normal field conditions. The field researchers did not hide the fact that they were doctors, which may have given them more authority than nurses. Solar radio installations allowed health centres in Ouallam to call for the district ambulance and surely lowered barriers for referral [48]. Since availability of emergency transport was not a problem in Ouallam, the difference between acceptance of the emergency referral and actual compliance was probably linked to the cost of the emergency transport. In any case, the large difference between acceptance and compliance rates suggests that the acceptance rate has little operational significance for monitoring. A combination of referral and compliance rate, however, provides a good indication for the performance of the referral system: low referral rates compared with a benchmark demonstrates general intake problems at the health centre level [46]. Low compliance points specifically to operational problems in the referral system. Both lead to -in principle- avoidable suffering and mortality.

Compliance rates for cold referrals were difficult to establish under the present study conditions. But they were likely to be equally low. At first sight this may be less dramatic than the referral reluctance for emergency cases. However, beside the possibility that conditions might complicate and need an emergency evacuation later, it is also significant in that it affects the credibility of the health centre: every non-respected referral is an unsatisfied patient with an expressed need but with an inadequate response of the health service.

At 1%, the referral rate for most of the rural health centres in Niger is well below the 2.5% benchmark. It seems to correspond with the exclusive referral of young adults with an emergency. Other referrals seem less acceptable or feasible for the staff, for the population or for both. As a consequence, hospital utilisation is generally very low. Niger invested significantly in district hospitals during the past 10 years in order to bring hospital care closer to the rural poor. The extremely low utilisation of these hospitals jeopardizes this investment. Even if they would offer the full range of care one can reasonably expect, district hospitals will start to play their life-saving role only when at the same time referral rates improve drastically. Making affordable emergency transport available to health centres should be a priority for any district in Niger and probably for many other parts of the world [48]. So far, Niger's health services have hardly invested in their referral systems. Health system managers were not aware of the problem and explained low hospital use through geographical inaccessibility. The health information system was unable to identify (and even less to quantify) a problem with referrals because without a benchmark, referral rate figures remain meaningless.

Setting a referral rate benchmark based on strict implementation of therapeutic guidelines makes it possible to monitor district performance in this area. When combined with information on compliance, it allows the assessment of initiatives to strengthen the referral system. Hospital utilisation data are no substitute for this because they reflect geographical and financial barriers as well as by-passing the first contact level.

## Conclusion

Establishing a referral benchmark allowed evaluating routine referral rates in rural health centres for their curative services. Health centres under routine conditions are seriously under referring. This leads to otherwise avoidable deaths. Especially children seem to pay a high tribute. IMCI proposed referral rates need revisiting.

The exercise proposes a low-cost tool for other districts in the world to evaluate their referral system for curative services. The proposed benchmark is context specific and can not be used by simple extrapolation.

## Competing interests

The author(s) declare that they have no competing interests.

## Authors' contributions

PB, RA, MSA, HM, A-M D and WVL were involved in the development of the concept and the data analysis and interpretation. PB, RA, MSA, HM organised and supervised the data collection in Niger. PB and RA trained the field researchers, PB, A-M D and WVL were in charge of the final write-up.

## Pre-publication history

The pre-publication history for this paper can be accessed here:



## Figures and Tables

**Table 1:** referral rates and proportion of emergency referrals

Data collection	New cases	Number of referrals proposed	Referral rate (%)	Number of emergencies	Percentage of emergencies among referrals
A (3 health centres, 150, 45 and 40 km from the hospital)	991 (in 3 months)	21	2.1	9	43%
B (1 health centre, 60 km from the hospital)	1169 (in 2 months)	28	2.4	17	61%
C (1 health centre, 150 km from the hospital)	1745 (in 3 months)	47	2.7	21	47%
**Total**	**3905**	**96**	**2.5**	**47**	**49%**

**Table 2:** Acceptance and compliance rates of referral proposals

Data collection	General referral acceptance (rate – %)	Cold referrals acceptance (rate – %)	Emergency referrals acceptance (rate – %)	Emergency referrals compliance (rate – %)
A	17 (81%)	9 (75%)	8 (89%)	5 (56%)
B	23 (82%)	11 (100%)	12 (71%)	9 (53%)
C	44 (94%)	24 (92%)	20 (95%	12 (57%)
**Total**	**84 (88%)**	**44 (90%)**	**39 (83%)**	**26 (55%)**

**Table 3:** Reasons for referral

Classification of pathology	Number	Proportion (%)
Gynaeco-obstetric	17	18%
Surgical	19	20%
Pediatric – non-surgical	19	20%
General medicine	41	42%

Total	96	100%
